# Effects of Vitexin, a Natural Flavonoid Glycoside, on the Proliferation, Invasion, and Apoptosis of Human U251 Glioblastoma Cells

**DOI:** 10.1155/2022/3129155

**Published:** 2022-03-02

**Authors:** Jinxiang Huang, Yini Zhou, Xinzhu Zhong, Fulai Su, Luning Xu

**Affiliations:** ^1^Department of Neurosurgery, Shanghai Institute of Neurosurger y, Shanghai Changzheng Hospital of Naval Medical University, Shanghai 200003, China; ^2^Department of Ophthalmology, Shanghai Jiao Tong University Affiliated Sixth People's Hospital, Shanghai 200233, China; ^3^Department of Pharmacy, Sanming First Hospital, Affiliated Hospital of Fujian Medical University, Sanming, 365000 Fujian Province, China

## Abstract

Glioblastoma is a highly aggressive brain tumor characterized by high recurrence and poor prognosis. Vitexin has shown activities against esophageal, liver, lung, colorectal, and ovarian cancers; however, there is little knowledge on the activity of vitexin against glioblastoma. This study was therefore designed with aims to examine the effects of vitexin on proliferation, invasion, and apoptosis of human U251 glioblastoma cells and explore the underlying molecular mechanisms using mRNA sequencing and molecular docking. Vitexin was found to inhibit cell proliferation, colony formation, and invasion and promote apoptosis in U251 cells. mRNA sequencing identified 499 differentially expressed genes in vitexin-treated U251 cells relative to controls, including 154 upregulated genes and 345 downregulated genes. Gene ontology (GO) term enrichment analysis revealed that the upregulated genes were most significantly enriched in intrinsic apoptotic signaling pathway and the downregulated genes were most significantly enriched in positive regulation of cell development and positive regulation of locomotion relating to biological processes, endoplasmic reticulum lumen and side of membrane relating to cellular components, and receptor ligand activity and receptor regulator activity relating to molecular functions. Kyoto Encyclopedia of Genes and Genomes (KEGG) pathway enrichment analysis revealed that the upregulated genes were involved in the pathways of transcriptional misregulation in cancer and the downregulated genes were involved in FoxO and JAK/STAT signaling pathways. Western blotting assay revealed that vitexin treatment resulted in reduced p-JAK1, p-JAK3, and p-STAT3 protein expression in U251 cells relative to untreated controls, and molecular docking predicted that vitexin had docking scores of –8.8, –10.8, and –10.5 kJ/mol with STAT3, JAK1, and JAK2, respectively. The results of the present study demonstrate that vitexin inhibits the proliferation and invasion and induces the apoptosis of glioblastoma U251 cells through suppressing the JAK/STAT3 signaling pathway, and vitexin may be a promising potential agent for the chemotherapy of glioblastoma.

## 1. Introduction

Glioma is the most common and also the most deadly primary intracranial tumor, representing approximately 80% of all malignant tumors in the central nervous system [1]. Although relatively rare, gliomas are associated with a high health burden in terms of significant morbidity and mortality [2]. Glioblastoma multiforme, the most aggressive form of gliomas, is characterized by high recurrence and high mortality [3] and has a 5-year survival rate of approximately 5% and a mean survival period of one year [4]. Currently, there is no cure for glioblastoma multiforme, and the treatment of this highly aggressive malignancy includes conventional surgical resection, radiotherapy, chemotherapy with temozolomide, or bevacizumab, as well as emerging gene therapy and immunotherapy; however, the overall response remains unsatisfactory [5–7]. A search for highly effective and lowly toxic agents is therefore of urgent need and of great significance to improve the prognosis among patients with glioblastoma multiforme [8].

Vitexin, a natural flavonoid compound extracted from the leaves and seeds of *Vitex negundo*, is abundant in a large number of edible and medicinal plants [9]. Vitexin has shown multiple pharmacological actions, including anti-inflammatory, antioxidative, neuroprotective, cardioprotective hepatoprotective, fat-inhibitory, and glucose-lowering activities [10, 11]. Previous studies have shown that vitexin suppresses the growth of esophageal, liver, lung, colorectal, and ovarian cancers [12, 13], and vitexin was found to inhibit the cell viability in a dose- and time-dependent manner and induce G2/M cell cycle arrest and apoptosis by inhibiting Akt/mTOR signaling in human glioblastoma LN-18 cells, suggesting that vitexin may serve as a therapeutic agent for treatment of malignant glioblastoma [14]. In addition, vitexin was identified as a phytochemical with significant alleviative effect on synthetic chemotherapy-induced toxicities [15]. It is therefore hypothesized that vitexin is a potential chemotherapeutic agent against human cancers, including glioblastoma. To test the hypothesis, this study was designed with aims to examine the effects of vitexin on biological behaviors of human U251 glioblastoma cells and explore the underlying mechanisms using mRNA sequencing and molecular docking, so as to provide insights into the development of novel agents for the treatment of glioblastoma multiforme.

## 2. Materials and Methods

### 2.1. Cell Line

The human U251 glioblastoma cell line was purchased from the Cell Bank of Type Culture Collection of Chinese Academy of Sciences (Shanghai, China) and incubated in Dulbecco's Modified Eagle Medium (DMEM) high-sugar medium (Hyclone; Logan, UT, USA) supplemented with 10% fetal bovine serum (FBS; Gibco, Grand Island, NY, USA) and 1% penicillin/streptomycin (Beijing Solarbio Science & Technology Co., Ltd.; Beijing, China) at 37°C containing 5% CO_2_. Cells were digested with 0.25% pancreatin and passaged at a ratio of 1 : 3. Log-phase cells were harvested for the subsequent experiments.

### 2.2. Chemicals

Vitexin (purity, ≥98%) was purchased from Chengdu Alfa Biotechnology Co., Ltd. (Chengdu, China). Temozolomide was purchased from Dalian Meilun Biology Technology Co., Ltd. (Dalian, China).

### 2.3. Cell Proliferation Assay

Log-phase U251 cells were prepared into single cell suspensions in DMEM supplemented with 10% FBS, seeded onto 96-well plates (Corning, Inc.; Corning, NY, USA) at a density of 1 × 10^4^ cells/well, of 100 *μ*L in each well, and incubated at 37°C for 24 hours. After cells grew to be adherent to the plate wall, vitexin was added at final concentrations of 0, 5, 10, 20, 40, 60, 100, 120, 180, 240, 360, and 480 *μ*M, and cells exposed to temozolomide at concentrations of 0, 3.125, 6.25, 12.5, 25, 50, 100, 150, 200, 250, and 300 *μ*M served as positive controls, while untreated cells served as negative controls, with triplicate wells assigned for each concentration. Following 48 h further incubation, 10 *μ*L Cell Counting Kit-8 (CCK-8) solutions (Beijing Solarbio Science & Technology Co., Ltd.; Beijing, China) were transferred to each well and incubated at 37°C containing 5% CO_2_ for one hour. Subsequently, the absorbance (*A* value) was read at 450 nm, and the half maximal inhibitory concentration (IC_50_) was estimated using the software GraphPad Prism version 8.0 (GraphPad Software, Inc.; La Jolla, CA, USA). Each assay was repeated in triplicate.

Log-phase U251 cells were prepared into single-cell suspensions in DMEM supplemented with 10% FBS, seeded onto 96-well plates at a density of 1 × 10^4^ cells/well, of 100 *μ*L in each well, and incubated at 37°C for 24 hours. After cells grew to be adherent to the plate wall, cells were exposed to vitexin or temozolomide at an IC_50_ concentration for further 72 hours, while untreated cells served as negative controls. Then, 10 *μ*L CCK-8 solutions were transferred to each well at 24, 48, and 72 hours and incubated for further one hour. Subsequently, the *A* value was read at 450 nm, and the cell proliferation curve was plotted.

### 2.4. Clonogenic Assay

Log-phase U251 cells were prepared into single cell suspensions in DMEM supplemented with 10% FBS, seeded onto 6-well plates (Corning, Inc.; Corning, NY, USA) at a density of 2 × 10^3^ cells/well and incubated at 37°C containing 5% CO_2_ for 24 hours. After growing to be adherent to the plate wall, cells were exposed to vitexin or temozolomide at an IC_50_ concentration for 2 days, and then, the culture medium was changed to DMEM supplemented with 10% FBS for further 2 to 3 weeks of incubation. The supernatant was discarded, and cells were gently rinsed twice in PBS (Beijing Solarbio Science & Technology Co., Ltd.; Beijing, China), fixed in 4% paraformaldehyde (Sinopharm Chemical Reagent Co., Ltd.; Shanghai, China) for 15 min, and the fixation solution was discarded. Cells were subsequently stained with 0.1% crystal violet (Beijing Solarbio Science & Technology Co., Ltd.; Beijing, China) for 10 to 30 min, rinsed with running water, and dried in air. The number of cell colony was counted using the software ImageJ version 1.44 (U.S. National Institutes of Health; Bethesda, MD, USA). Each assay was repeated in triplicate.

### 2.5. Transwell Invasion Assay

Matrigel matrix (BD Biosciences; Franklin Lakes, NJ, USA) was diluted with DMEM at a ratio of 1 : 5, and Transwell chambers were immersed in PBS for 5 min to make moist, coated with 80 *μ*L Matrigel matrix, and solidified in an incubator at 37°C for 30 min. Log-phase U251 cells were prepared into single-cell suspensions in DMEM supplemented with 10% FBS and gently centrifuged, and cells were harvested, resuspended in serum-free medium containing vitexin or temozolomide at an IC_50_ concentration and adjusted to a density of 1.0 × 10^5^ cells/mL. Then, 100 *μ*L cell suspensions were transferred to the upper chamber, and the lower chamber was added with 500 *μ*L medium supplemented with 20% FBS for further 48-hour incubation. Transwell chambers were removed, and the medium was discarded. Cells that did not migrate from upper Transwell chambers were gently removed with cotton swabs. Cells were washed twice in calcium-free PBS, fixed in 4% formaldehyde for 30 min, dried in air, stained with 0.1% crystal violet for 20 min, washed three times in PBS, and photographed under a microscope. The number of cells was counted using the software ImageJ version 1.44. Each assay was repeated in triplicate.

### 2.6. Apoptosis Assay

Log-phase U251 cells were seeded onto 6-well plates and treated with vitexin or temozolomide at an IC_50_ concentration for 48 hours after cells grew to 70% confluence. Cells were then digested with EDTA-free pancreatin, centrifuged at 300 × *g* for 5 min at 4°C. The sediment was harvested, washed in PBS, centrifuged at 300 × *g* for 5 min at 4°C. The sediment was harvested, resuspended in 100 *μ*L staining buffer, and incubated with 5 *μ*L Annexin V-FITC binding buffer and 5 *μ*L propidium iodide (PI) solution for 10 min at room temperature. The cell apoptosis was detected using flow cytometry. Each assay was repeated in triplicate.

### 2.7. Western Blotting

U251 cells treated with vitexin at a concentration at an IC_50_ concentration and untreated cells were incubated for 48 hours and lysed in RIPA Lysis and Extraction Buffer (Beyotime Biotechnology; Shanghai, China) containing protease inhibitor Cocktail. The protein concentration was quantified using the BCA Protein Assay Kit (Beyotime Biotechnology; Shanghai, China). Total protein was separated on SDS-PAGE and electrotransferred to nitrocellulose (NC) membranes (Millipore, Billerica, MA, USA). NC membranes were blocked in 5% nonfat milk (Beijing Solarbio Science & Technology Co., Ltd.; Beijing, China) for one hour, washed three times in TBST, and incubated with rabbit anti-human STAT3 monoclonal antibody (1 : 1,000; Cell Signaling Technology, Inc., Danvers, MA, USA), rabbit anti-human phospho-STAT3 monoclonal antibody (1 : 1,000; Cell Signaling Technology), mouse anti-human JAK1 monoclonal antibody (1 : 1,000; Cell Signaling Technology), rabbit anti-human phospho-JAK1 monoclonal antibody (1 : 1,000; Cell Signaling Technology), rabbit anti-human JAK2 monoclonal antibody (1 : 1,000; Abcam, Waltham, MA, USA), and rabbit anti-human phospho-JAK2 monoclonal antibody (1 : 1,000; Abcam), while GAPDH (1 : 2,000; Proteintech Group, Inc., Rosemont, IL, USA) served as a loading control. Then, the immunoblots were incubated with HPR-conjugated goat anti-rabbit secondary antibody (1 : 1,0000; Beyotime Biotechnology, Shanghai, China) at 37°C for one hour, stained with enhanced ECL chemiluminescent substrate (Millipore; Billerica, MA, USA), and visualized with the Tanon 5200 Chemiluminescent Imaging System (Tanon Science & Technology Co., Ltd.; Shanghai, China). The gray density of each protein band was normalized to that of GAPDH. Each assay was repeated in triplicate.

### 2.8. mRNA Sequencing

Total RNA was extracted from U251 cells treated with vitexin at a concentration at an IC_50_ concentration and untreated cells using the TRIzol reagent kit (Thermo Fisher Scientific; Carlsbad, CA, USA). The RNA purity was measured using the NanoDrop One/Onec Spectrophotometer (NanoDrop Technologies, Wilmington, USA), and the RNA concentration was quantified using the Qubit® 3.0 Fluorometer (Life Technologies; Carlsbad, CA, USA), while the RNA integrity was evaluated using the Agilent 4200 TapeStation system (Agilent Technologies; Santa Clara, CA, USA). Eligible total RNA samples were used for mRNA purification on magnetic beads containing Oligo (dT). The first-strand cDNA was synthesized with mRNA fragments as a template and random oligonucleotides as a primer in the M-MuLV reverse transcriptase system, and the RNA strand was degraded with RNaseH. The second-strand cDNA was synthesized in the DNA polymerase I system, and the double-stranded cDNA was purified, followed by end repair, dA-tailing, and adaptor ligation. The cDNA library was screened with AMPure XP beads to yield cDNA with 200 bp in size. Then, PCR was performed, and the amplification products were purified using AMPure XP beads (Beckman Coulter, Inc.; Brea, CA, USA). The library concentration and size were detected using KAPA qPCR assay [16] and the Agilent 4200 TapeStation system. Eligible library was sequenced on a Hiseqxten Pe150 Illumina Sequencing Platform (Illumina, Inc.; San Diego, CA, USA). Original sequencing data were processed using the fastp software [17], for gene expression analysis and differentially expressed gene analysis. In addition, the differentially expressed genes were subject to Gene ontology (GO) term enrichment analysis and Kyoto Encyclopedia of Genes and Genomes (KEGG) enrichment analyses using the clusterProfiler software [18].

In addition, eligible total RNA samples were transcribed into cDNA, followed by quantitative real-time PCR assay to quantify the relative gene expression using the specific primers (Table 1), with *18S* as an internal control. The relative gene expression was estimated using the 2^-∆∆Ct^ method.

### 2.9. Molecular Docking

The three-dimensional (3D) structures of target STAT3 (PDB ID: 6NJS), JAK1 (PDB ID: 6BBU), and JAK2 proteins (PDB ID: 3KRR) were downloaded from the RSCB PDB database (http://www.rcsb.org/pdb/home/home.do) and saved as PDB-formatted files. The two-dimensional (2D) structure of vitexin was retrieved in the PubChem Substance and Compound database (https://pubchem.ncbi.nlm.nih.gov/), format-converted using the Open Babel toolbox and saved as mol2-formatted files. All preprocessed target proteins and original ligands were saved as PDB-formatted files. Then, the layout of the grid box and grid volume was done using the software AutoDockTools version 1.5.6, and all active small molecules, preprocessed target proteins, and original ligands were converted to pdbqt-formatted files. The AutoDockTools software was run to dock active small molecules and target proteins. Finally, the molecular docking results were visualized using the Pymol software. A docking score of <0 indicates free binding of vitexin to the target protein of glioblastoma, and a lower score represents a high likelihood of the interaction between vitexin and the target protein of glioblastoma.

### 2.10. Statistical Analysis

All measurement data were described as the mean ± standard deviation (SD), and comparisons of means between groups were done using Student's *t* test. All categorical data were expressed as proportions, and differences were proportions were tested for statistical significance with a chi-square test. All statistical analyses were performed using the software GraphPad Prism version 8.0, and a *P* value < 0.05 was considered statistically significant.

## 3. Results

### 3.1. Vitexin Suppresses U251 Cell Proliferation and Invasion and Promotes Cell Apoptosis

**Figure 1:**
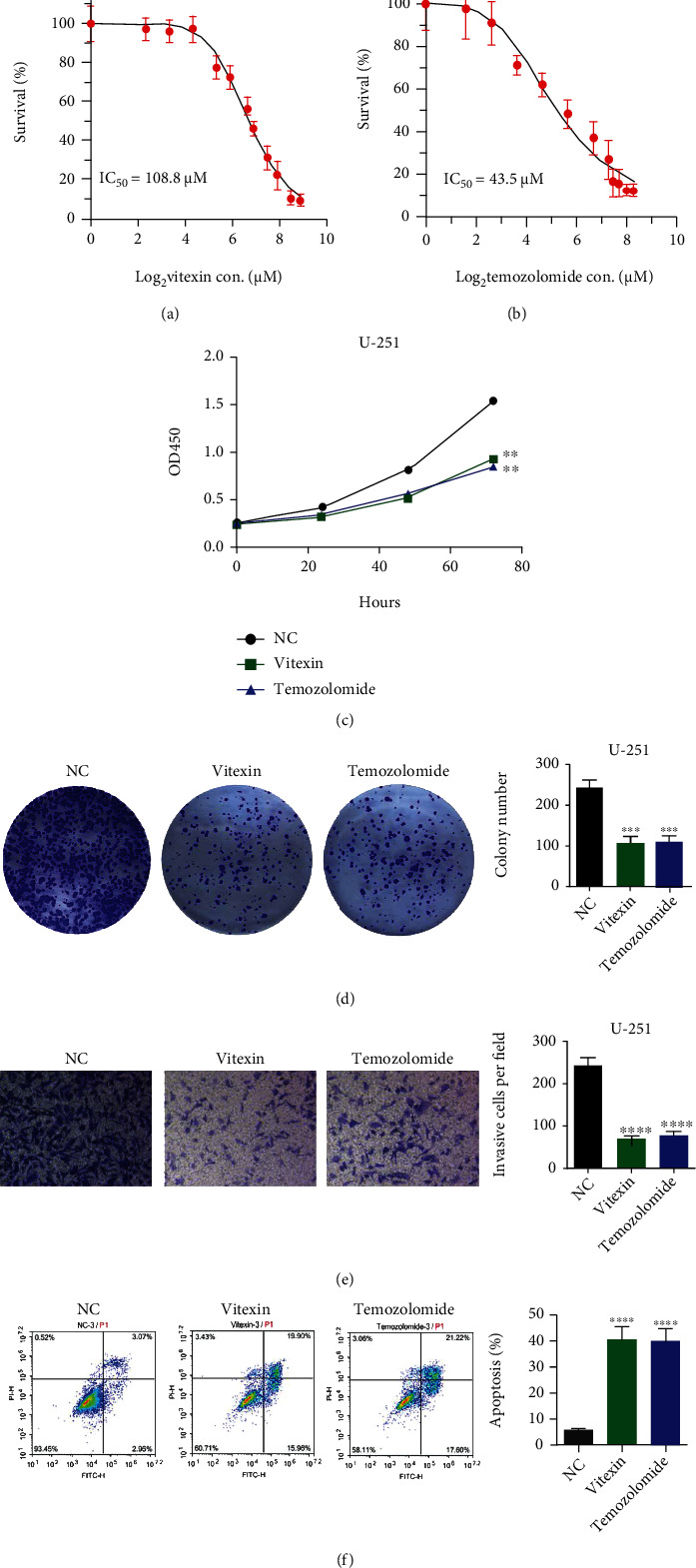
Effects of vitexin on proliferation, cell colony formation, invasion, and apoptosis of U251 cells. (a, b) CCK-8 assay measures the IC_50_ values of vitexin and temozolomide against U251 cells. (c) CCK-8 assay detects the effect of vitexin on U251 cell proliferation, with temozolomide as a positive control. (d) Clonogenic assay detects the effect of vitexin on U251 cell colony formation, with temozolomide as a positive control. (e) Transwell invasion assay detects the effect of vitexin on U251 cell invasion, with temozolomide as a positive control. (f) Flow cytometry detects the effect of vitexin on apoptosis of U251 cells, with temozolomide as a positive control. Each bar represents the mean ± SD from three independent assays. ^∗∗^*P* < 0.01;  ^∗∗∗^*P* < 0.001;  ^∗∗∗∗^*P* < 0.0001.

To examine the inhibition of vitexin on U251 cells, we first measured the vitexin IC_50_ values against U251 cells, with temozolomide serving as a positive control. CCK-8 assay showed 108.8 and 34.5 *μ*M IC_50_ values of vitexin and temozolomide for U251 cells, respectively (Figures 1(a) and 1(b)). Exposure to vitexin at an IC_50_ concentration remarkably suppressed U251 cell proliferation (Figure 1(c)) and inhibited cell colony formation (Figure 1(d)) and invasion (Figure 1(e)). In addition, flow cytometry detected a higher apoptotic rate in U251 cells treated with vitexin at an IC50 concentration than untreated cells (Figure 1(f)).

### 3.2. Effects of Vitexin on Transcriptomic Profiles of U251 Cells

**Figure 2:**
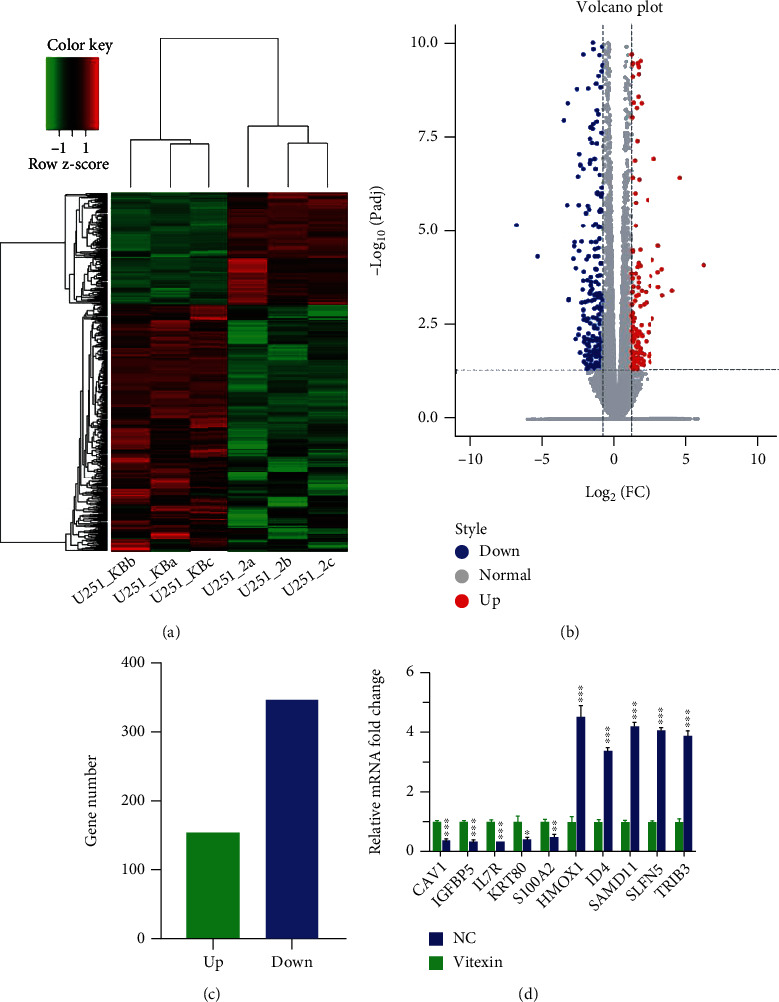
Effects of vitexin on transcriptomic profiles of glioblastoma U251 cells. (a) Volcano plot of differentially expressed genes. (b) Heat map of differentially expressed gene. (c) Number of differentially expressed genes. (d) qPCR assay quantifies the expression of 5 randomly selected upregulated and 5 randomly selected downregulated genes as revealed by mRNA sequencing. Each bar represents the mean ± SD from three independent assays. ^∗∗^*P* < 0.01;  ^∗∗∗^*P* < 0.001.

To unravel the molecular mechanisms underlying the effect of vitexin on biological functions of glioblastoma, mRNA sequencing was performed to examine the effects of vitexin on transcriptomic profiles of glioblastoma U251 cells, and 499 differentially expressed genes were identified in vitexin-treated U251 cells relative to untreated cells, including 154 upregulated genes and 345 downregulated genes (Figures 2(a)–2(c)).  Table 2 shows the 20 most differentially expressed genes. Then, 5 upregulated genes and 5 downregulated genes were randomly selected for further validation using qPCR assay, and the expression of these 10 genes was consistent with the transcriptomic analysis (Figure 2(d)), indicating the reliability of mRNA sequencing.

### 3.3. GO Term Enrichment Analysis of Differentially Expressed Genes

**Figure 3:**
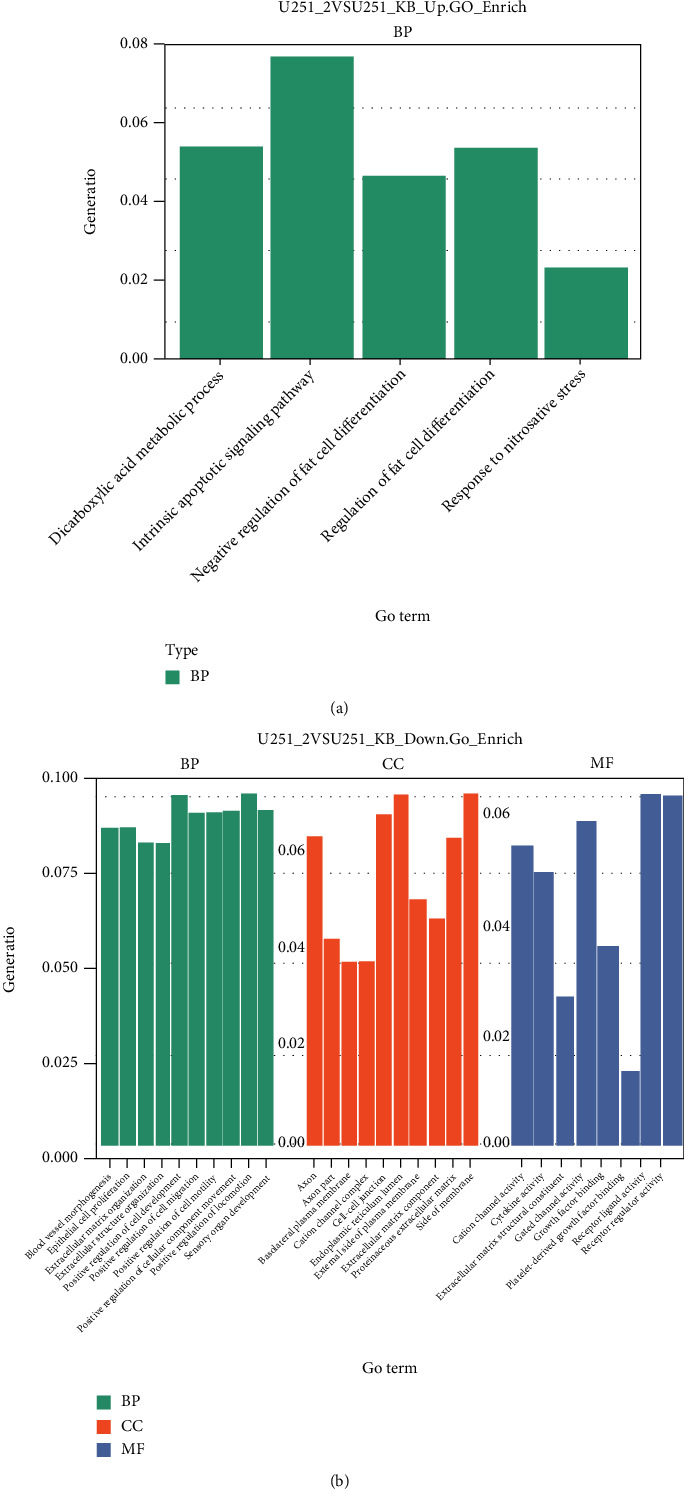
GO term enrichment analysis of differentially expressed genes between vitexin-treated and untreated U251 cells. (a) GO term enrichment analysis of upregulated genes. (b) GO term enrichment analysis of downregulated genes.

Then, we predicted the functions of differentially expressed genes in U251 cells following exposure to vitexin, and GO term enrichment analysis revealed that the upregulated genes were most significantly enriched in intrinsic apoptotic signaling pathway (GO: 0097193 under biological process, *P* = 0.027) (Figure 3(a)), and the downregulated genes relating to biological process, cellular components, and molecular functions were most significantly enriched in positive regulation of cell development (GO: 0010720 under biological process, *P* = 0.0003), positive regulation of locomotion (GO: 0040017 under biological process, *P* = 0.0003), endoplasmic reticulum lumen (GO: 0005788 under cellular components, *P* = 0.0002), side of membrane (GO: 0098552, *P* = 0.0059), receptor ligand activity (GO: 0048018 under molecular function, *P* = 0.0003), and receptor regulator activity (GO: 0030545 under molecular function, *P* = 0.0008) (Figure 3(b)). In addition, significant enrichment of cell-cell junction (GO: 0005911 under cellular components, *P* = 0.0103), cytokine activity (GO: 0005125 under molecular function, *P* = 0.005), extracellular matrix structural constituent (GO: 0005201 under molecular function, *P* = 0.0006), and growth factor binding (GO: 0019838 under molecular function, *P* = 0.005) was seen in downregulated genes (Figure 3(b)).

### 3.4. KEGG Pathway Enrichment Analysis of Differentially Expressed Genes

**Figure 4:**
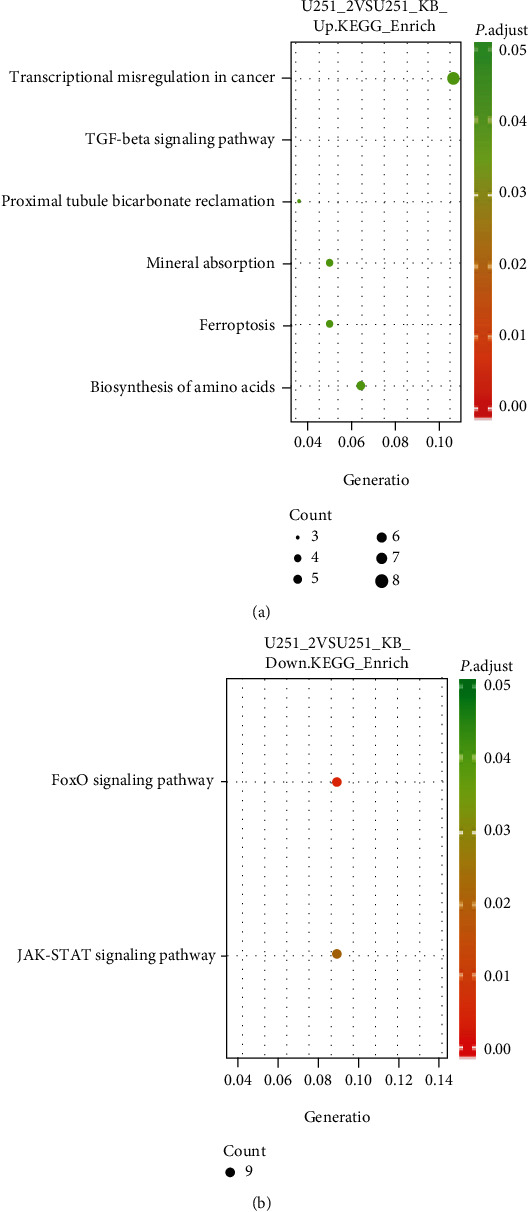
KEGG pathway analysis of differentially expressed genes between vitexin-treated and untreated U251 cells. (a) Bubble chart of upregulated genes. (b) Bubble chart of downregulated genes.

KEGG pathway enrichment analysis revealed that the upregulated genes were involved in 6 pathways, with transcriptional misregulation in cancer (hsa05202, *P* = 0.038) as the most significant pathway, and the 345 downregulated genes were involved in only two significant pathways, FoxO (hsa04068, adjusted, *P* = 0.0093) and JAK-STAT signaling pathways (hsa0463, adjusted, *P* = 0.0226) (Figure 4).

### 3.5. Effects of Vitexin on JAK/STAT Signaling Pathway in U251 Cells

**Figure 5:**
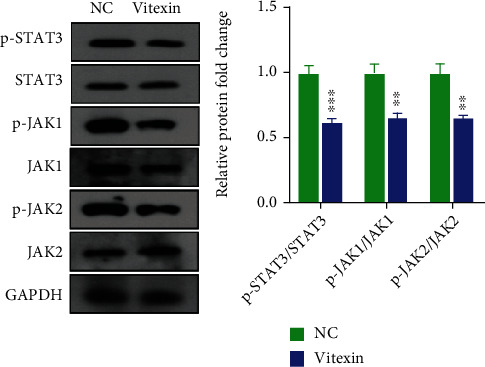
Western blotting determines the effect of vitexin on JAK/STAT signaling pathway in U251 cells. U251 cells were treated with vitexin at a concentration of 108.8 *μ*M or DMSO for 48 hours, and then, cells were harvested. Total protein was extracted from cells and subjected to Western blotting assay. Each bar represents the mean ± SD from three independent assays. ^∗∗^*P* < 0.01;  ^∗∗∗^*P* < 0.001.

Since KEGG pathway enrichment analysis revealed downregulation of JAK/STAT signaling pathway-associated genes in vitexin-treated U251 cells, it was therefore hypothesized that vitexin might inhibit glioblastoma growth and proliferation through suppressing the JAK/STAT signaling. To test our hypothesis, Western blotting assay was performed to quantify the expression of JAK/STAT signaling-associated proteins and phosphorylated proteins in vitexin-treated and untreated U251 cells. We determined significantly lower p-JAK1, p-JAK2, and p-STAT3 expression in vitexin-treated U251 cells than in untreated cells (Figure 5), suggesting vitexin inhibits the activity of the JAK/STAT signaling pathway.

### 3.6. Molecular Docking Prediction

**Figure 6:**
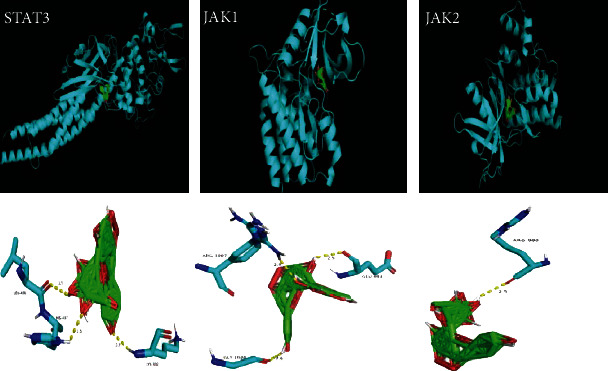
Three-dimensional molecular docking of vitexin binding to JAK1, JAK2, and STAT3 proteins.

If vitexin served as ligand, and STAT3 (6NJS), JAK1 (6BBU), and JAK2 (3KRR), the critical target proteins of the JAK/STAT signaling pathway, as receptors, vitexin was predicted to have docking scores of –8.8, –10.8, and –10.5 kJ/mol, indicating that vitexin binds stably to STAT3, JAK1, and JAK2. In addition, vitexin bound to the amino acid residues of STAT3, including Leu-436, His-437, and Lys-370 to form multiple hydrogen bonds with a high affinity, and vitexin was found to be embedded in the JAK1 protein cavity to bind to the amino acid residues of JAK1, including Arg-1007, Gly-1020, and Glu-883, to form multiple hydrogen bonds, which increased the structural stability. Moreover, vitexin was found to bind to the Arg-980 amino acid site in JAK2 protein (Figure 6). These data demonstrate that vitexin has a high affinity of binding to the critical target proteins in the JAK/STAT signaling pathway.

## 4. Discussion

Glioblastoma multiforme is a highly aggressive brain tumor that is associated with high mortality [1]. More importantly, the incidence of glioblastoma multiforme is on a rise across the world [19]. With the increasing understanding of the molecular mechanisms of glioblastoma multiforme pathogenesis, reversion of the malignant phenotype of glioblastoma cells through targeted blockade of major signaling pathways involved in development, progression and chemotherapeutic resistance of glioblastoma multiforme has been paid much attention [20, 21].

JAK/STAT signaling, a common pathway for multiple cytokines/growth factors signaling, is widely involved in cell proliferation, differentiation and apoptosis, and inflammation [22]. Seven STAT family members have been identified until now, including STAT1, STAT2, STAT3, STAT4, STAT5A, STAT5B and STAT6, and STAT3 and STAT5 are found to promote cell proliferation and transformation and suppress apoptosis [23]. Elevated levels of STAT3 tyrosine phosphorylation have been detected in glioma specimens or cells, indicating activation of STAT3 [24–26], and glioma patients with a high proportion of STAT3 phosphorylation-positive cells were found to have shorter survival than those with a low proportion [27]. It has been shown that inhibition of STAT3 activity results in suppression of antiapoptotic proteins (Survivin, Bcl-xL, and Bcl-2) and cell cycle-regulating proteins (c-Myc, cyclin D1, and cyclin E) expression [28], and ascochlorin was found to suppress glioma cell migration and invasion through inhibiting MMP-2 expression by targeting the JAK2/STAT signaling pathway [29]. In addition, glioma stem cells were reported to activate the STAT3 signaling through secreting IL-6 and IL-10, thereby resulting in activation of B7-H4 expression in tumor-associated macrophages [30], while B7-H4 triggered the escape of glioma-initiating cells from immune surveillance in the microenvironment of gliomas through blocking effective T-cell immune responses [31], indicating that STAT3 is involved in immune escape of gliomas and promotes glioma progression.

Vitexin is a flavonoid compound extracted from the natural medicinal plant *V. negundo* [9]. Previous studies have shown that vitexin to cytotoxic to liver, colorectal, oral, esophageal, lung, ovarian, and leukemia cancer cells through promoting cancer cell apoptosis and autophagy and inhibiting cell proliferation and migration via multiple pathways [10–13, 32, 33]. Vitexin was found to abrogate hepatocellular carcinoma invasion and angiogenesis and induce hepatocellular carcinoma cell apoptosis through suppressing AKT/STAT3 signaling or activating JNK signaling [34–36], and it has been reported that vitexin inhibits proliferation and triggers apoptosis of glioblastoma, non-small-cell lung cancer, and renal cell carcinoma cells through suppressing the PI3K/Akt/mTOR signaling pathway [14, 37, 38]. In addition, vitexin has shown inhibition against nasopharyngeal carcinoma, colorectal carcinoma, epithelial ovarian cancer, and leukemia by targeting NF-*κ*B, JNK, ERK, and RAS/RAF pathways, respectively [39–42]. In the current study, vitexin was found to suppress glioblastoma U251 cell proliferation and invasion and promote cell apoptosis, which was similar to the previous report [14]. Unlike the findings from Zhang and colleagues reporting that vitexin treatment resulted in inhibition of the Akt/mTOR signaling pathway in human glioblastoma LN-18 cells [14], the results of the present study showed a reduction in the activity of the JAK/STAT signaling pathway following vitexin treatment, which may be attributed to different cell lines.

Previous studies have shown that vitexin treatment leads to apoptosis, a reduction in mitochondrial membrane potential and Bcl-2 protein expression and elevated Caspase-3 and Caspase-9 protein expression in human non-small-cell lung cancer A549 cells and human leukemia K-562 cells [37, 42]. In this study, GO term enrichment analysis showed that the upregulated genes were most significantly enriched in intrinsic apoptotic signaling pathway, suggesting that vitexin may trigger glioblastoma U251 cell apoptosis via the mitochondria/cytochrome c-mediated intrinsic apoptotic signaling pathway. It was reported that vitexin protected human ECV-304 endothelial cells against tertbutyl hydroperoxide- (TBHP-) induced injury and alleviated the toxicity of glutamate and amyloid *β*-protein against mouse neuroblastoma Neuro-2a cells through increasing mitochondrial membrane potential [43, 44]. Although the impact of vitexin on mitochondrial membrane potential has not been fully understood until now, these data imply that mitochondria may be an important target organelle for pharmaceutical actions exerted by vitexin.

As a common pathway for multiple cytokine/growth factor signaling, STAT proteins respond to the activation signals transferred by the binding of cytokines and receptors and are activated by growth factor receptors, such as epidermal growth factor receptor (EGFR) and platelet-derived growth factor receptor (PDGFR) [22]. In the current study, GO term enrichment analysis showed that the downregulated genes were significantly enriched in cytokine activity, receptor ligand activity, platelet-derived growth factor binding, receptor regulator activity, and growth factor binding, which was in agreement with our *in vitro* assays showing that vitexin suppressed glioblastoma U251 cell proliferation and invasion. In addition, our GO term enrichment analysis revealed that the downregulated genes were significantly enriched in positive regulation of cell motility, positive regulation of cell migration, and epithelial cell proliferation, although glioblastoma is not derived from epithelial cells.

It has been demonstrated that aberrant activation of the STAT3 protein plays a critical role during glioma development and progression, such as promoting angiogenesis and immune escape of gliomas and glioma cell proliferation and differentiation [45]. With the advances in understanding of the JAK/STAT3 signaling in gliomas, it is gradually accepted that STAT3 is the junction site of multiple signaling pathways [45], and therefore, targeting the STAT3 signaling pathway has emerged as a promising therapeutic strategy for numerous cancers [46]. Knockdown of STAT3 expression by RNAi was found to suppress growth and induce apoptosis and differentiation in glioblastoma stem cells [47], and WP1006, a selective inhibitor of JAK, was shown to be inhibitory against malignant glioma cell growth both *in vitro* and *in vivo* [48]. In this study, molecular docking predicted that vitexin had docking scores of –8.8, –10.8, and –10.5 kJ/mol with STAT3, JAK1, and JAK2, suggesting that vitexin binds stably to the critical proteins in the JAK/STAT3 signaling pathway and vitexin may be a potential inhibitor of the JAK/STAT3 signaling pathway.

## 5. Conclusions

In summary, the results of the present study demonstrate that vitexin inhibits the proliferation and invasion and induces the apoptosis of glioblastoma U251 cells through suppressing the JAK/STAT3 signaling pathway. It is therefore considered that vitexin may be a promising potential agent for the chemotherapy of glioblastoma. Further studies to test the activity of vitexin against glioblastoma in animal models seem justified.

## Figures and Tables

**Table 1:** Sequences of primers used for qPCR assay.

Gene name	Primer sequence
*SLFN5*	F: 5′-GCAGGAAAAGTCACCCTTGGG-3′ R: 5′-CAGAGCACATACTGCTCGCA-3′
*HMOX1*	F: 5′-ACAAGGAGAGCCCAGTCTTCG-3′ R: 5′-CGCTTCACATAGCGCTGCAT-3′
*TRIB3*	F: 5′-AAGCGGTTGGAGTTGGATGAC-3′ R: 5′-CACGATCTGGAGCAGTAGGTG-3′
*SAMD11*	F: 5′-ACCTCGTTATGCCCGAGCAT-3′ R: 5′-AAGCAGTCGCTGCTGATACG-3′
*ID4*	F: 5′-CTGCCGCTCTTCGTCTACT-3′ R: 5′-GAACCTGTCCACGCTGATG-3′
*IL7R*	F: 5′-TGTCGTCTATCGGGAAGGAG-3′ R: 5′-CGGTAAGCTACATCGTGCATTA-3′
*IGFBP5*	F: 5′-GCCAAGAGGGCGACAAGTT-3′ R: 5′-GCTGCCCATCAGCTTCTTCAG-3′
*CAV1*	F: 5′-TGACCGCAAAGGATTCTACAAG-3′ R: 5′-CGTCAACGTACTCCATGCCT-3′
*KRT80*	F: 5′-GCGACCCTAAACACCTCAAC-3′ R: 5′-ATGCCGTCAAAACTGTGTGTC-3′
*18S*	F: 5′-CGACGACCCATTCGAACGTCT-3′ R: 5′-CTCTCCGGAATCGAA CCCTGA-3′

**Table 2:** The 20 most differentially expressed genes.

Upregulated gene		Downregulated gene	
Gene name	log_2_ fold change	Gene name	log_2_ fold change
*CCL20*	5.96	*miR-205HG*	–6.83
*AC007923.1*	4.33	*ELF3*	–5.4
*SLC12A7*	3.85	*PADI3*	–4.33
*TATDN2P3*	3.17	*KRT13*	–4.12
*CYP4F11*	3.14	*LINC01405*	–3.71
*CXCL11*	2.9	*PARP15*	–3.61
*SGCG*	2.89	*RTL3*	–3.38
*DHRS2*	2.60	*APOBEC3G*	–3.33
*CXCL10*	2.47	*DIRC3*	–3.31
*LINC01583*	2.47	*CYB5R2*	–3.28
*CHAC1*	2.47	*PSG5*	–3.27
*SESN2*	2.35	*ZNF488*	–3.04
*ATP1A3*	2.29	*CDH3*	–2.95
*CHRNA9*	2.29	*AC008440.3*	–2.89
*NMRAL2P*	2.28	*HHIPL1*	–2.86
*VGLL2*	2.28	*MS4A3*	–2.8
*KLF15*	2.27	*KIRREL3*	–2.79
*TRIB3*	2.24	*PADI1*	–2.77
*PPP2R2C*	2.21	*IL7R*	–2.7
*ID4*	2.18	*GRIN2B*	–2.7

## Data Availability

All data presented in this study are available upon request by contact with the corresponding author.

## References

[1] Weller M., Wick W., Aldape K. (2015). Glioma. *Nature Reviews. Disease Primers*.

[2] GBD 2016 Brain and Other CNS Cancer (2019). Global, regional, and national burden of brain and other CNS cancer, 1990-2016: a systematic analysis for the Global Burden of Disease Study 2016. *The Lancet Neurology*.

[3] Batash R., Asna N., Schaffer P., Francis N., Schaffer M. (2017). Glioblastoma multiforme, diagnosis and treatment; recent literature review. *Recent Literature Review. Curr Med Chem.*.

[4] Holland E. C. (2000). Glioblastoma multiforme: the terminator. *Proceedings of the National Academy of Sciences of the United States of America*.

[5] Stoyanov G. S., Dzhenkov D., Ghenev P., Iliev B., Enchev Y., Tonchev A. B. (2018). Cell biology of glioblastoma multiforme: from basic science to diagnosis and treatment. *Medical Oncology*.

[6] Anjum K., Shagufta B. I., Abbas S. Q. (2017). Current status and future therapeutic perspectives of glioblastoma multiforme (GBM) therapy: a review. *Biomedicine & Pharmacotherapy*.

[7] Sasmita A. O., Wong Y. P., Ling A. P. K. (2018). Biomarkers and therapeutic advances in glioblastoma multiforme. *Asia-Pacific Journal of Clinical Oncology*.

[8] Shergalis A., Bankhead A., Luesakul U., Muangsin N., Neamati N. (2018). Current challenges and opportunities in treating glioblastoma. *Pharmacological Reviews*.

[9] Peng Y., Gan R., Li H. (2021). Absorption, metabolism, and bioactivity of vitexin: recent advances in understanding the efficacy of an important nutraceutical. *Critical Reviews in Food Science and Nutrition*.

[10] He M., Min J. W., Kong W. L., He X. H., Li J. X., Peng B. W. (2016). A review on the pharmacological effects of vitexin and isovitexin. *Fitoterapia*.

[11] Gu C. B., Cai M., Yuan X. H., Zu Y. G. (2015). Research progress on plant resources distribution of vitexin and its pharmacological effects. *Zhongguo Zhong Yao Za Zhi*.

[12] Ganesan K., Xu B. (2017). Molecular targets of vitexin and isovitexin in cancer therapy: a critical review. *Annals of the New York Academy of Sciences*.

[13] Zhou Y., Liu Y. E., Cao J. (2009). Vitexins, nature-derived lignan compounds, induce apoptosis and suppress tumor growth. *Clinical Cancer Research*.

[14] Zhang G., Li D., Chen H., Zhang J., Jin X. (2018). Vitexin induces G2/M-phase arrest and apoptosis via Akt/mTOR signaling pathway in human glioblastoma cells. *Molecular Medicine Reports*.

[15] Farzaei M. H., Bahramsoltani R., Rahimi R. (2016). Phytochemicals as adjunctive with conventional anticancer therapies. *Current Pharmaceutical Design*.

[16] Mardis E., McCombie W. R. (2017). *Library quantification using SYBR green-quantitative polymerase chain reaction (qPCR)*.

[17] Chen S., Zhou Y., Chen Y., Gu J. (2018). fastp: an ultra-fast all-in-one FASTQ preprocessor. *Bioinformatics*.

[18] Yu G., Wang L. G., Han Y., He Q. Y. (2012). clusterProfiler: an R package for comparing biological themes among gene clusters. *OMICS*.

[19] Grech N., Dalli T., Mizzi S., Meilak L., Calleja N., Zrinzo A. (2020). Rising incidence of glioblastoma multiforme in a well-defined population. *Cureus*.

[20] Bryukhovetskiy I., Bryukhovetskiy A., Khotimchenko Y., Mischenko P. (2016). Novel cellular and post-genomic technologies in the treatment of glioblastoma multiforme (review). *Oncology Reports*.

[21] Bambury R. M., Morris P. G. (2014). The search for novel therapeutic strategies in the treatment of recurrent glioblastoma multiforme. *Expert Review of Anticancer Therapy*.

[22] Harrison D. A. (2012). The Jak/STAT pathway. *Cold Spring Harbor Perspectives in Biology*.

[23] Kisseleva T., Bhattacharya S., Braunstein J., Schindler C. W. (2002). Signaling through the JAK/STAT pathway, recent advances and future challenges. *Gene*.

[24] Mizoguchi M., Betensky R. A., Batchelor T. T., Bernay D. C., Louis D. N., Nutt C. L. (2006). Activation of STAT3, MAPK, and AKT in malignant astrocytic gliomas: correlation with EGFR status, tumor grade, and survival. *Journal of Neuropathology and Experimental Neurology*.

[25] Rahaman S. O., Harbor P. C., Chernova O., Barnett G. H., Vogelbaum M. A., Haque S. J. (2002). Inhibition of constitutively active Stat3 suppresses proliferation and induces apoptosis in glioblastoma multiforme cells. *Oncogene*.

[26] Schaefer L. K., Ren Z., Fuller G. N., Schaefer T. S. (2002). Constitutive activation of Stat3*α* in brain tumors: localization to tumor endothelial cells and activation by the endothelial tyrosine kinase receptor (VEGFR-2). *Oncogene*.

[27] Birner P., Toumangelova-Uzeir K., Natchev S., Guentchev M. (2010). STAT3 tyrosine phosphorylation influences survival in glioblastoma. *Journal of Neuro-Oncology*.

[28] Wu N., Liu J., Zhao X. (2015). Cardamonin induces apoptosis by suppressing STAT3 signaling pathway in glioblastoma stem cells. *Tumour Biology*.

[29] Cho H. J., Park J. H., Nam J. H., Chang Y. C., Park B., Hoe H. S. (2018). Ascochlorin suppresses MMP-2-mediated migration and invasion by targeting FAK and JAK-STAT signaling cascades. *Journal of Cellular Biochemistry*.

[30] Borisov K. E., Sakaeva D. D. (2015). The immunosuppressive microenvironment of malignant gliomas. *Arkhiv Patologii*.

[31] Yao Y., Ye H., Qi Z. (2016). B7-H4 (B7x)-mediated cross-talk between glioma-initiating cells and macrophages via the IL6/JAK/STAT3 pathway lead to poor prognosis in glioma patients. *Clinical Cancer Research*.

[32] Yang S. H., Liao P. H., Pan Y. F., Chen S. L., Chou S. S., Chou M. Y. (2013). The novel p53-dependent metastatic and apoptotic pathway induced by vitexin in human oral cancer OC2 cells. *Phytotherapy Research*.

[33] Lee C. Y., Chien Y. S., Chiu T. H. (2012). Apoptosis triggered by vitexin in U937 human leukemia cells via a mitochondrial signaling pathway. *Oncology Reports*.

[34] Wang J., Zheng X., Zeng G., Zhou Y., Yuan H. (2014). Purified vitexin compound 1 inhibits growth and angiogenesis through activation of FOXO3a by inactivation of Akt in hepatocellular carcinoma. *International Journal of Molecular Medicine*.

[35] Lee J. H., Mohan C. D., Shanmugam M. K. (2020). Vitexin abrogates invasion and survival of hepatocellular carcinoma cells through targeting STAT3 signaling pathway. *Biochimie*.

[36] He J. D., Wang Z., Li S. P. (2016). Vitexin suppresses autophagy to induce apoptosis in hepatocellular carcinoma via activation of the JNK signaling pathway. *Oncotarget*.

[37] Liu X., Jiang Q., Liu H., Luo S. (2019). Vitexin induces apoptosis through mitochondrial pathway and PI3K/Akt/mTOR signaling in human non-small cell lung cancer A549 cells. *Biological Research*.

[38] Li Y., Sun Q., Li H., Yang B., Wang M. (2020). Vitexin suppresses renal cell carcinoma by regulating mTOR pathways. *Translational Andrology and Urology*.

[39] Wang W., Cheng H., Gu X., Yin X. (2019). The natural flavonoid glycoside vitexin displays preclinical antitumor activity by suppressing NF-*κ*B signaling in nasopharyngeal carcinoma. *Oncotargets and Therapy*.

[40] Bhardwaj M., Paul S., Jakhar R. (2017). Vitexin confers HSF-1 mediated autophagic cell death by activating JNK and ApoL1 in colorectal carcinoma cells. *Oncotarget*.

[41] Zhao S., Guan X., Hou R. (2020). Vitexin attenuates epithelial ovarian cancer cell viability and motility *in vitro* and carcinogenesis *in vivo* via p38 and ERK1/2 pathways related VEGFA. *Ann Transl Med.*.

[42] Sarkar M. K., Kar A., Jayaraman A., Kar Mahapatra S., Vadivel V. (2022). Vitexin isolated from *Prosopis cineraria* leaves induce apoptosis in K-562 leukemia cells via inhibition of the BCR-ABL-Ras-Raf pathway. *The Journal of Pharmacy and Pharmacology*.

[43] Li H. B., Ying X. X., Lu J. (2010). The mechanism of vitexin-4″-O-glucoside protecting ECV-304 cells against tertbutyl hydroperoxide induced injury. *Natural Product Research*.

[44] Malar D. S., Prasanth M. I., Shafreen R. B., Balamurugan K., Devi K. P. (2018). _Grewia tiliaefolia_ and its active compound vitexin regulate the expression of glutamate transporters and protect Neuro-2a cells from glutamate toxicity. *Life Sciences*.

[45] Atkinson G. P., Nozell S. E., Benveniste E. T. (2010). NF-kappaB and STAT3 signaling in glioma: targets for future therapies. *Expert Review of Neurotherapeutics*.

[46] Zou S., Tong Q., Liu B., Huang W., Tian Y., Fu X. (2020). Targeting STAT3 in cancer immunotherapy. *Molecular Cancer*.

[47] Li G. H., Wei H., Lv S. Q., Ji H., Wang D. L. (2010). Knockdown of STAT3 expression by RNAi suppresses growth and induces apoptosis and differentiation in glioblastoma stem cells. *International Journal of Oncology*.

[48] Iwamaru A., Szymanski S., Iwado E. (2007). *A novel inhibitor of the STAT3 pathway induces apoptosis in malignant glioma cells both _in vitro_ and _in vivo_*. *Oncogene*.

